# Association between Plasmatic Ceramides Profile and AST/ALT Ratio: C14:0 Ceramide as Predictor of Hepatic Steatosis in Adolescents Independently of Obesity

**DOI:** 10.1155/2017/3689375

**Published:** 2017-05-28

**Authors:** Jorge Maldonado-Hernández, Gabriela E. Saldaña-Dávila, Mónica I. Piña-Aguero, Benjamín A. Núñez-García, Mardia G. López-Alarcón

**Affiliations:** Laboratorio de Espectrometría de Masas, Unidad de Investigación Médica en Nutrición, Hospital de Pediatría, Centro Médico Nacional Siglo XXI, IMSS, Ciudad de México, Mexico

## Abstract

**Objective:**

To assess the association between plasma ceramides and hepatic steatosis (HS) in adolescents, independently of obesity.

**Materials and Methods:**

Ninety-four adolescents from two previous studies conducted and published by our crew were included. Study subjects were stratified in three groups: normal weight (*n* = 18), obesity (*n* = 34), and obesity + HS (*n* = 42). The presence of HS was defined when ALT/AST ratio was <1. Ceramides subspecies (C14:0, C16:0, C18:0, C24:0, and C24:1) were determined by LC/MS.

**Results:**

All ceramides correlated directly with ALT levels and inversely with ALT/AST ratio; the strongest correlation was observed among C14:0 ceramide (*r* = 0.41 and *r* = −0.54, resp.; *P* < 0.001). Furthermore, significant correlations were observed between cholesterol and all ceramides except for C24:1 ceramide. Interestingly ceramides C14:0, C18:0, and C24:1 correlated directly with both fasting insulin and HOMA-IR index. For assessing HS, a cut-off point of 10.3 nmol/L for C14:0 ceramide reported a sensitivity of 92.7% and a specificity of 73.5% when normal weight and obesity groups (*n* = 52) were compared against obesity + HS group (*n* = 42). Positive and negative predictive values were 77.5% and 90.2%, respectively.

**Conclusions:**

Plasma ceramides are closely associated with hepatic steatosis in adolescents. C14:0 ceramide could be a novel biomarker of HS independently of obesity.

## 1. Introduction

Ceramides (Cer) belongs to the sphingolipid family and are formed by a sphingosine and a fatty acid group linked by an amide bond. Typically, the fatty acid moiety is 14 to 26 carbon atoms' chain length, most of them saturated or monounsaturated. Ceramides are produced in the organism by three different pathways: (1) the novo synthesis from palmitate and serine in four sequential reactions in the endoplasmic reticulum, (2) from the hydrolysis of plasma membrane sphingomyelin via sphingomyelinase (SMase), and (3) a salvage pathway from sphingosine that can be recycled back to ceramide [1]. Several isoforms of SMase that can be distinguished by their optima pH, neutral SMase (NSMase), and acid SMase (ASMase) are mostly found in mitochondria and lysosomes, respectively [2].

It has been described that during obesity there is an accumulation of several ceramides species in human tissues as muscle, adipose tissue, liver, and heart. Obesity leads to the activation of inflammatory signals that promote ceramides production [3, 4]. It is well known that TNF-*α* increases ceramides production via SMase that hydrolyses sphingomyelin, the most abundant sphingolipid specie in cell membranes [5]. Ceramides accumulation has been involved in insulin signaling mechanisms that induce insulin resistance in muscle and adipose tissue [6–8]. In the liver, in addition to glucose impairment metabolism, ceramides have been implicated in the induction of hepatic steatosis [9].

Most of the studies that analyzed the role of ceramides and their relationship with hepatic steatosis (HS) have been conducted in animal models. It has been hypothesized that ceramides could have a role as second messengers in the development and progression of nonalcoholic fatty liver disease (NAFLD) through mechanisms that promote oxidative stress, inflammation, and apoptosis [9, 10]. The liver is an important site of ceramides production due to an increased free fatty acid flux as observed in higher adiposity and inflammation settings. It has been estimated that 80% of plasma ceramides are present in both hepatic lipoproteins: VLDL and LDL [11]. The aspartate aminotransferase (AST)/alanine transaminase (ALT) ratio is a useful screening biomarker of hepatic steatosis. Serum AST and ALT concentrations increase with body weight, although it is more prominent for ALT. Metabolic disturbances as insulin resistance, dyslipidemia, and hyperglycemia are closely related to elevated ALT; a cut-off point of AST/ALT ratio <1 has been defined for hepatic steatosis assessment in obese patients [12].

Global prevalence of NAFLD in children is estimated in 3% and could increase up to 40% in the presence of obesity [13]. Ceramides could play an important role in the pathogenesis of this disease, although clinical studies in pediatric population are limited and the role of ceramides is not well established. The aim of this study was to describe plasma ceramides profile in adolescents with and without hepatic steatosis and evaluate the association between specific ceramides and ALT/AST ratio, independently of obesity as well as assess if any of the analyzed ceramides could be an adequate biomarker to predict hepatic steatosis.

## 2. Materials and Methods

### 2.1. Study Population

Clinical data and biological samples of ninety-four adolescents from two previous studies conducted and published by our crew were selected by convenience [14, 15]. Only those subjects who had intact plasma for liver enzymes and ceramides determinations were included. Subjects of both studies were enrolled during 2011 and 2012 in Mexico City, Mexico. Both studies complied with the World Medical Association Declaration of Helsinki regarding ethical conduct of research in human subjects. Protocols were approved by the Ethics Committee of the Mexican Institute of Social Security with register numbers R-2010-3603-35 [14] and R-2010-3603-14 [15], respectively. Informed consent was obtained from the representative of all the study subjects.

### 2.2. Biochemical Determinations

Peripheral blood samples were obtained after 10–12 hours of overnight-fast in Vacutainer test tubes with and without EDTA. Samples were centrifuged at 3000 rpm during 10 minutes. Plasma and serum were preserved at −70°C until analysis. Concentrations of fasting glucose, triglycerides, HDL, and VLDL were previously determined with commercial kits in an automated spectrophotometer Spin 120 (Spinreact Girona, Spain; coefficients of variation ~3.9%). Fasting insulin was determined by radioimmunoassay kits (Millipore, Billerica, MA) with coefficient of variation (CV) of 7.5%.

AST and ALT concentrations were determined in intact serum samples preserved all the time at −70°C, with enzymatic-colorimetric kits in a Spin 120 spectrophotometer.

### 2.3. Plasma Ceramides Extraction and Quantification

An adaptation of the method described by Kasumov et al. was performed [16]. All solvents used in the following description were of chromatographic grade. First, 1.4 mL of methanol was added to 200 *μ*L of blood plasma to precipitate the proteins. Then the sample was centrifuged at 3500 rpm for 10 minutes. Once the phases were separated, the supernatant was decanted in a new test tube and 1.2 mL of chloroform was added. Mixture was vortexed for one minute; afterwards 1 mL of water was added and vortexed for another minute. Sample was centrifuged once again at 3500 rpm for 10 minutes. The aqueous phase (upper layer) was discarded and the organic phase (bottom) was evaporated with ultra high purity nitrogen at 40°C. The dried extract was reconstituted with 500 *μ*L of dichloromethane and passed through a Strata® SI-1 Silica column (55 *μ*m, 70 Å, 200 mg/3 mL) (Phenomenex; California, USA) previously washed with 500 *μ*L of dichloromethane. Ceramides were eluted 3 times with 500 *μ*L of dichloromethane/isopropanol solution (70 : 30, v/v). The elution was lead to evaporation with ultra high pure nitrogen. The extract was reconstituted with 200 *μ*L of ethanol and stored at −70°C. Samples were analyzed by LC-MS on a Waters Xevo TQD Acquity UPLC H-Class (Waters; Milford, USA) equipment with a LC Column 50 × 21 mm Kinetex® 2.6 *μ*m C8 100 Å (Phenomenex; California, USA). The column was preheated at 50°C, the flow was established at 0.4 mL/min, and the total run time was of 18 minutes. The aqueous phase (A) was prepared with ultrapure water and formic acid at 0.2%; organic phase (B) was a mixture of acetonitrile and 2-propanol (60 : 40, v/v) and 0.2% of formic acid. Ceramides species were resolved with a gradient starting from 40% mobile phase A and 60% of mobile phase B during 0.2 minutes to 100% phase B over 13.8 minutes at linear gradient and then 100% B for 0.5 min. Mass spectrometer capillary voltage was 3.9 kV, cone voltage 40 V, and the source temperature 150°C.

Ceramide external standards with a d18:1 sphingoid base (C14:0, C16:0, C18:0, C24:0, and C24:1 subspecies; Avanti Polar Lipids, Inc., Alabaster, Alabama, USA) were diluted with 1 mL of CHCl_3_ in order to obtain a 5 mg/mL concentration. A “Standard Mix” containing the 5 ceramides was prepared at a concentration of 1000 ng/mL (1 *μ*g/mL) of each ceramide, in a total volume of 25 mL in ethanol. Several dilutions were prepared to create a calibration curve with the following concentrations: 1000 ng/mL, 500 ng/mL, 250 ng/mL, 125 ng/mL, 62.5 ng/mL, 31.25 ng/mL, and 7.81 ng/mL. The coefficient of variation for the LC-MS analysis was <6.5%. Ceramides data was converted to nmol/L.

### 2.4. Statistical Analysis

Data were analyzed using the Statistical Package for the Social Studies (SPSS version 20, SPSS Inc., Chicago, IL, USA). Kolmogorov-Smirnov test was used to assess data distribution. Biochemical results are expressed as median (percentile 25–percentile 75); comparison among study groups was performed with Mann–Whitney *U* test. Plasmatic ceramides concentrations are expressed as mean (confidence interval 95%); comparison between study groups was made by ANOVA test with Tukey post hoc analysis. Correlation between plasmatic ceramides and biochemical features was performed with a Spearman's correlation analysis. A receiver operator characteristic (ROC) curve was constructed to determine an optimal cut-off point for a specific ceramide to predict hepatic steatosis. Area Under the Curve (AUC) with 95% confidence interval (CI) was obtained; positive and negative predictive values were determined.

## 3. Results

Ninety-four adolescents were included in the present study; 41 were females (43.6%) and 53 males (56.4%). According to BMI and AST/ALT ratio, subjects were stratified in 3 groups: normal weight group (P15th to P85th of the WHO Child Growth Standards [17] and AST/ALT ratio > 1); obesity group (*P* > 95th and AST/ALT ratio > 1); and obesity group with hepatic steatosis (*P* > 95th and AST/ALT ratio < 1). Biochemical characteristics of the subjects are shown in Table 1. Significant differences were found between normal weight group and obesity group in almost all variables except for AST, cholesterol, and fasting glucose levels. A similar behavior was observed when comparing normal weight group and obesity group + HS but, additionally, no significant difference was found in HDL levels. Interestingly, when comparing obesity group and obesity + HS group we just found significant differences in BMI, ALT levels, and AST/ALT ratio.

**Figure 1:**
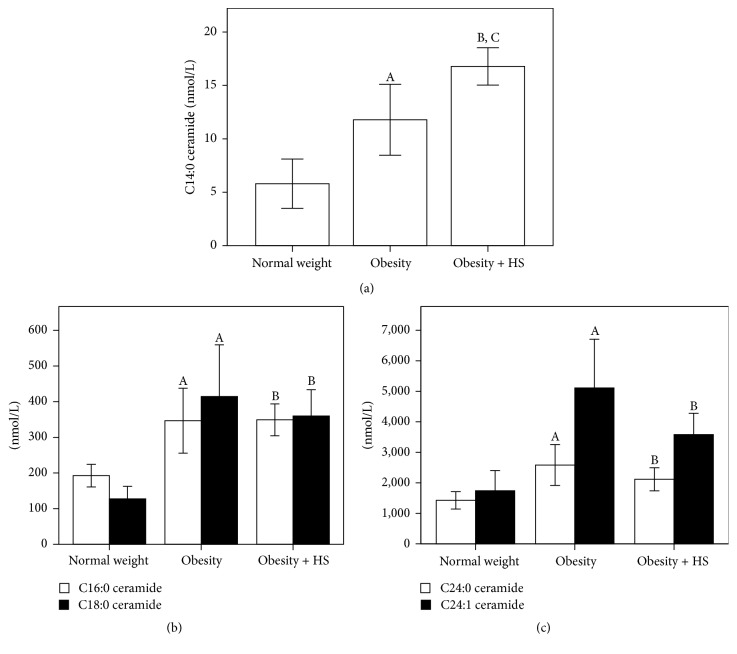
Comparison of ceramides subspecies among study groups. ^A^Significant difference (*P* < 0.05) between obesity and normal weight group; ^B^between obesity + HS and normal weight group; ^C^between obesity + HS and obesity group.

Plasma ceramides profile stratified by study groups are shown in Figure 1. For C14:0 ceramide (C14:0 Cer) significant differences were observed when comparing normal weight group versus obesity and obesity + HS groups. Likewise, obesity + HS group had significantly higher levels of C14:0 Cer than obesity group. For the other ceramides, significantly higher levels were observed in obesity and obesity + HS groups when comparing to normal weight subjects, although no significance was observed when comparing obesity group versus obesity + HS group.

Spearman's correlation coefficient between specific ceramides and biochemical markers of hepatic function, dyslipidemia, and insulin resistance surrogates are shown in Table 2. All ceramides correlated directly with ALT levels and inversely with AST/ALT ratio; the strongest correlation was observed among C14:0 Cer. Furthermore, significant correlations were observed between cholesterol and all ceramides except for C24:1 subspecies. Interestingly ceramides C14:0, C18:0, and C24:1 correlated directly with both fasting insulin and HOMA-IR index.

**Figure 2:**
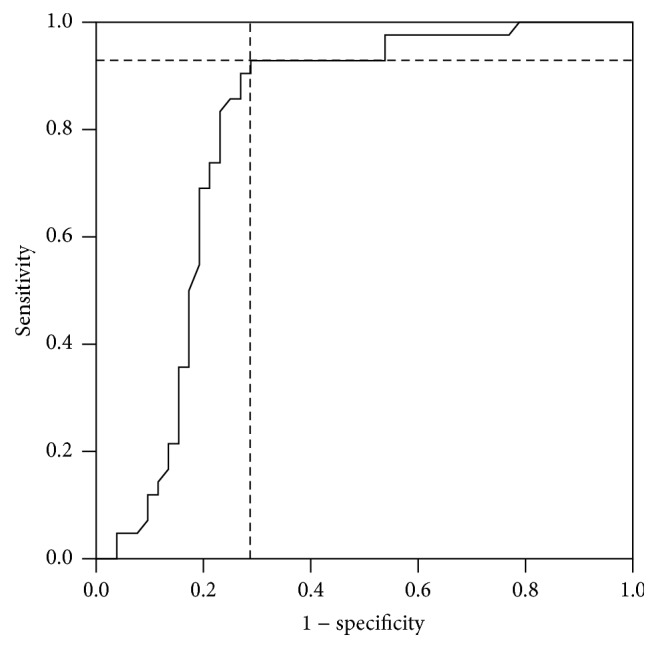
Receiver operator characteristic (ROC) curve for C14:0 ceramide. ROC curve was computed contrasting data from normal weight and obesity groups (*n* = 52) versus obesity group with HS (*n* = 42).

Finally, a ROC analysis was performed to determine an optimal cut-off point for a specific ceramide as an adequate biomarker of hepatic steatosis. C14:0 Cer reported the best attributes to predict HS. ROC curve was computed contrasting data from normal weight and obesity groups (*n* = 52) versus obesity + HS group (*n* = 42).  Figure 2 shows the ROC curve obtained; a cut-off point of 10.3 nmol/L for C14:0 Cer had a sensitivity of 92.7% and a specificity of 73.5% (AUC = 80.4%, *P* < 0.001; 95% CI = 70.2–90.5%). Positive and negative predictive values were 77.5% and 90.2%, respectively.

## 4. Discussion

The present study demonstrated that hepatic steatosis is closely related to plasmatic ceramides concentration. All the subspecies analyzed correlated inversely with AST/ALT ratio and directly with ALT levels. Although, only C14:0 ceramide was significantly different among the three study groups, no differences were observed in the other analyzed ceramides between obesity group and obesity + HS group. C14:0 ceramide proved to be the better predictor of HS in comparison with the other ceramides analyzed with adequate diagnosis features, independently of obesity. Interestingly, Tomita et al. found similar results to ours for serum-free myristic acid (C14:0) to predict nonalcoholic steatohepatitis (NASH) with a sensitivity and a specificity of 57.1% and 85%, respectively. Positive and negative predictive values were in turn 93.6% and 34.0% [18]. The C14:0 ceramide analyzed in this study have had enhanced diagnostic attributes than myristic acid, probably because liver is a key site of ceramides production and is therefore a more specific indicator of what happens in liver tissue [9]. Serum-free fatty acids may be reflecting global lipid metabolism that may be influenced by diet, *β*-oxidation, and lipid mobilization. On the other hand, a strength of the study of Tomita et al. is that NASH was established by histological assessment in liver biopsy.

In addition to the correlations observed among ALT and the AST/ALT ratio, the examined ceramides also correlated with higher levels of cholesterol (except C24:1 Cer) and C14:0, C18:0, and C24:1 ceramides were directly associated with fasting insulin and HOMA-IR index. Recently, Kasumov et al. found that C14:0 ceramide is related to insulin sensitivity improvement after a 12-week exercise training program. A decrease in plasma levels of C14:0 ceramide was associated with changes in insulin-stimulated glucose disposal (*r* = −0.56; *P* < 0.01) in adult subjects with obesity or type 2 diabetes (T2D). It is well known that ceramides are associated with insulin resistance, although most of the published works have been done in adult population. There are few studies that have described the association between plasma ceramides, insulin resistance, and other metabolic disorders in pediatric population. Lopez et al. found that children and adolescents with T2D have higher concentrations of different subspecies of ceramides in comparison with lean healthy subjects [19].

A limitation of our study could be the use of the AST/ALT ratio as diagnostic criteria to establish the presence of fatty liver in the studied population. The* Ritis *ratio is not the gold standard for hepatic steatosis assessment and there are more precise methods for its diagnosis, such as liver biopsy or imaging studies as ultrasound, tomography, or magnetic resonance [12]. However, these techniques are not available for us and the retrolective character of this study did not allow us to use any of the above methods. It is therefore likely that the presence of fatty liver will become inconsistent particularly in some of the subjects in the groups categorized as “obesity” and “obesity + HS.” Regardless, the association found among the subspecies of ceramides and hepatic steatosis markers are independent of this stratification and it might even be more likely that a better delimitation of hepatic steatosis in the study subjects could enhance diagnostic attributes of C14:0 ceramide to predict the disease.

The increase of FFA flux towards the liver tissue observed in states of obesity and inflammation could be increasing the synthesis of ceramides and other lipid species in the liver. Locally, ceramides increase mitochondrial generation of reactive oxygen species that promote apoptosis and hepatic inflammation. The increase of FFA flux ratio overcomes the capacity of the liver to oxidize lipids so its exportation through low density lipoproteins could be a strategy of the liver to decrease the hepatotoxic effect of them. Since liver seems to be a key site for ceramides production, plasma ceramides levels could be a reflection of hepatic disturbances and a new goal for intervention studies to prevent the development of more serious liver diseases.

## 5. Conclusion

In summary, plasma ceramides are closely associated with hepatic steatosis in adolescents. As far as we know this is the first study to demonstrate that C14:0 ceramide could be a novel biomarker of HS independently of obesity.

## Figures and Tables

**Table 1:** Biochemical characteristics of study subjects.

	Normal weight (*n* = 18)	Obesity (*n* = 34)	Obesity + HS (*n* = 42)
Gender (female/male)	8/10	17/17	16/26
Age (years)	14 (12.75–15)	12 (11–13)^a^	13.5 (12–15)^b^
BMI (kg/m^2^)	20 (19–20.6)	26.8 (25.1–30.8)^a^	30.1 (27.5–36.1)^b,c^
AST (U/L)	31 (27–38.5)	39 (29.5–47.5)	37.5 (25–56)
ALT (U/L)	19 (16–22.5)	31 (21.5–45.5)^a^	54.5 (33.5–79.5)^b,c^
Ratio (AST/ALT)	1.7 (1.4–1.8)	1.3 (1–1.5)^a^	0.7 (0.6–0.8)^b,c^
Triglycerides (mg/dL)	82 (62.8–98.3)	114 (103.5–218)^a^	135.5 (92–179.3)^b^
Cholesterol (mg/dL)	151.5 (133.5–171)	159 (145.5–187.5)	161 (144.8–185.3)
HDL (mg/dL)	54.5 (48–60)	46 (41–51.5)^a^	47.5 (38–54)
VLDL (mg/dL)	16 (12.5–19.5)	23 (21–44)^a^	29 (18–36)^b^
Fating glucose (mg/dL)	84 (78–86)	87 (81–93.5)	87.5 (82.75–93)^b^
Fasting insulin (*μ*U/mL)	9.6 (7.5–14.7)	18.4 (15.7–22.2)^a^	19 (14.9–25.1)^b^
HOMA-IR	2.03 (1.6–2.9)	4.11 (3.2–4.8)^a^	4.12 (3.2–5.4)^b^

Data are presented as median (percentile 25–percentile 75). Comparison between groups was made with a Mann–Whitney *U* test. ^a^Significant difference (*P* < 0.05) between obesity and normal weight group; ^b^between obesity + HS and normal weight group; ^c^between obesity + HS and obesity group. HOMA-IR: Homeostasis Model Assessment for Insulin Resistance.

**Table 2:** Correlation matrix between different ceramides and biochemical features.

	C14:0 Cer	C16:0 Cer	C18:0 Cer	C24:0 Cer	C24:1 Cer
AST	0.085	0.045	0.12	0.06	0.08
ALT	0.41^*∗∗∗*^	0.21^*∗∗*^	0.34^*∗∗∗*^	0.19^§^	0.3^*∗∗*^
AST/ALT ratio	−0.54^*∗∗∗*^	−0.34^*∗∗∗*^	−0.36^*∗∗∗*^	−0.21^*∗*^	−0.33^*∗∗∗*^
Triglycerides	0.25^*∗*^	0.12	0.17	0.13	0.17
Cholesterol	0.25^*∗*^	0.28^*∗∗*^	0.28^*∗∗*^	0.3^*∗∗*^	0.16
HDL	−0.062	−0.11	−0.13	−0.31	−0.31^*∗∗*^
VLDL	0.305^*∗∗*^	0.16	0.21^*∗*^	0.15	0.21^§^
Fasting glucose	0.062	−0.09	−0.02	−0.15	0.01
Fasting insulin	0.21^*∗*^	0.19^§^	0.27^*∗∗*^	0.17^§^	0.31^*∗∗*^
HOMA-IR	0.21^*∗*^	0.15	0.23^*∗*^	0.1	0.25^*∗*^

Data correspond to Spearman correlation coefficients. ^*∗∗∗*^Significant  *P* values ≤ 0.001; ^*∗∗*^*P* ≤ 0.01; ^*∗*^*P* ≤ 0.05. ^§^Statistical trend (0.05 ≥ *P* < 0.1).
